# Decorin—An Antagonist of TGF-β in Astrocytes of the Optic Nerve

**DOI:** 10.3390/ijms22147660

**Published:** 2021-07-17

**Authors:** Magdalena Schneider, Andrea E. Dillinger, Andreas Ohlmann, Renato V. Iozzo, Rudolf Fuchshofer

**Affiliations:** 1 Institute of Human Anatomy and Embryology, University of Regensburg, 93053 Regensburg, Germany; magdalena.schneider@ur.de (M.S.); Andrea.Dillinger@vkl.uni-regensburg.de (A.E.D.); 2 Department of Ophthalmology, Ludwig-Maximilians-University Munich, 80336 Munich, Germany; andreas.ohlmann@med.uni-muenchen.de; 3 Department of Pathology, Anatomy, and Cell Biology, and the Translational Cellular Oncology Program, Sidney Kimmel Medical College at Thomas Jefferson University, Philadelphia, PA 19107, USA; renato.iozzo@jefferson.edu

**Keywords:** glaucoma, astrocytes, decorin, TGF-beta, reactive gliosis, optic nerve head, CTGF/CCN2, AKT signaling

## Abstract

During the pathogenesis of glaucoma, optic nerve (ON) axons become continuously damaged at the optic nerve head (ONH). This often is associated with reactive astrocytes and increased transforming growth factor (TGF-β) 2 levels. In this study we tested the hypothesis if the presence or absence of decorin (DCN), a small leucine-rich proteoglycan and a natural inhibitor of several members of the TGF family, would affect the expression of the TGF-βs and connective tissue growth factor (CTGF/CCN2) in human ONH astrocytes and murine ON astrocytes. We found that DCN is present in the mouse ON and is expressed by human ONH and murine ON astrocytes. DCN expression and synthesis was significantly reduced after 24 h treatment with 3 nM CTGF/CCN2, while treatment with 4 pM TGF-β2 only reduced expression of *DCN* significantly. Conversely, DCN treatment significantly reduced the expression of *TGF-β1*, *TGF-β2* and *CTGF/CCN2* vis-a-vis untreated controls. Furthermore, DCN treatment significantly reduced expression of fibronectin (*FN*) and collagen IV (*COL IV*). Notably, combined treatment with DCN and triciribine, a small molecule inhibitor of protein kinase B (AKT), attenuated effects of DCN on *CTGF/CCN2*, *TGF-β1*, and *TGF-β2* mRNA expression. We conclude (1) that DCN is an important regulator of *TGF-β* and *CTGF/CCN2* expression in astrocytes of the ON and ONH, (2) that DCN thereby regulates the expression of extracellular matrix (ECM) components and (3) that DCN executes its negative regulatory effects on *TGF-β* and *CTGF/CCN2* via the pAKT/AKT signaling pathway in ON astrocytes.

## 1. Introduction

Glaucoma, the second leading cause of blindness worldwide, is a neurodegenerative disorder due to a progressive degeneration of the optic nerve (ON) [1,2]. Until now, up to 127 gen loci associated with glaucoma could be identified in genome-wide association studies, showing a strong genetic predisposition to this disease [3,4]. Additionally, prospective randomized multicenter studies have identified intraocular pressure (IOP) as the main critical risk factor for onset and progression of primary open-angle glaucoma (POAG), the most frequent form of glaucoma [5,6,7,8,9]. In its progression, axonal transport is impaired due to the effects of mechanical stress induced by IOP on the peripapillary sclera and the optic nerve head (ONH), causing the injury and death of retinal ganglion cells (RGC) [10].

In a healthy eye the ON axons are supported by astrocytes to cope with diurnal IOP fluctuations and thereby with mechanical stress at the ONH. Astrocytes are vital for RGC health by forming an essential part of the blood-brain and blood-retinal barrier, by suppling neurons with nutrients and by maintaining the homeostatic balance of the extracellular environment [11]. Astrocytes influence neuronal tissue by restructuring the extracellular matrix (ECM) and releasing growth factors, cytokines and other cellular mediators under normal and pathological conditions [12,13,14,15,16].

During the pathogenesis of POAG, astrocytes in the ONH show definite signs of astrogliosis, including thickened processes and hypertrophy of the cell body [17,18]. ONH astrocytes are strongly involved in the remodeling of ECM, which is a typical process observed in POAG patients [19,20,21,22,23]. Many studies have shown that astrocytes of glaucomatous ONHs produce increased amounts of ECM, including Collagen Type IV (COL IV), Fibronectin (FN) or Laminin (LAM), while proteoglycans, such as e.g., aggrecan, are less synthesized [13,14,24,25,26].

Gene expression analysis identified transforming growth factor-β (TGF-βs) to be among the key molecules related to the pathological processes occurring in the glaucomatous ONH [13,27]. TGF-β2 is the predominant isoform in the ONH and is synthesized by neuroglial cells [28]. TGF-β2 has been found to be elevated in the ONH of POAG patients, it is predominantly expressed by reactive astrocytes [28,29] and causes increased expression and synthesis of ECM proteins [29,30,31]. These effects are mediated by connective tissue growth factor (CTGF/CCN2) in human ONH astrocytes [30]. Under normal conditions TGF-β2 is under a tight control of a molecular network that influences its activation, binding to its receptor and interfering with the signaling pathways.

We have recently discovered that decorin (DCN) is a key modulator of the TGF-β2 and CTGF/CCN2 activities in the outflow tissues. DCN was initially described as a regulator of collagen fibrillogenesis [32,33,34], but like other small leucine-rich proteoglycans, the biological function of DCN extends beyond the interaction with collagens. The biological effect of DCN was discovered by its abilities to interact with growth factors and receptor tyrosine kinases as well [35,36,37,38]. A vital role of DCN within the eye was proven as a mutation in the DCN gene causes congenital stromal corneal dystrophy [39,40] and lack of DCN leads to a glaucomatous phenotype in DCN-deficient mice, which fits to the observations that DCN is found in reduced amounts in the aqueous humor and in the outflow tissues of POAG patients [32,41,42]. Since the ON and ONH undergo changes during the course of POAG, which are most likely due to elevated levels of TGF-β and CTGF/CCN2, we tested the hypothesis if the presence or absence of DCN would also affect the expression of the *TGF-β*s and *CTGF/CCN2* in the ON and ONH.

In the present study, we investigated the influence of DCN on ON and ONH astrocyte biology. We discovered that DCN is produced by human ONH astrocytes and murine astrocytes of the ON and that DCN deficiency leads to an upregulation of *TGF-β* and *CTGF/CCN2* in the murine ON. Hence, we discovered reciprocal effects of TGF-β2, CTGF/CCN2 and DCN in these cell types. While we found that TGF-β2 and CTGF/CCN2 reduce the expression of *DCN*, we also could prove that DCN negatively regulates *TGF-β2* and *CTGF/CCN2* via the pAKT/AKT pathway and that DCN is able to reduce expression of the ECM components *FN* and *COL IV* in human ONH astrocytes and murine ON astrocytes. Thus, we propose that DCN could attenuate the remodeling process in the ONH occurring during the pathogenesis of glaucoma.

## 2. Results

### 2.1. Expression of Growth Factors Is Increased in the ON of DCN-Deficient Mice

**Figure 1:**
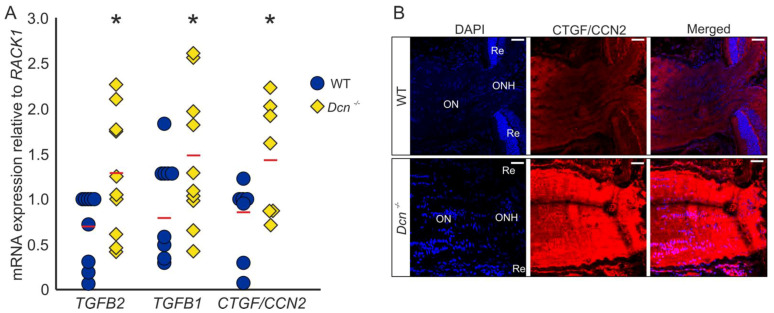
DCN deficiency increases the expression and synthesis of TGF-β1, -β2 and CTGF/CCN2 in the murine ON. (**A**) Expression of *TGF-β2* (WT = 1 ± 0.13, *n* = 9, *Dcn*^−/−^ = 1.85 ± 0.23, *n* = 10, *p* = 0.036), *TGF-β1* (WT = 1 ± 0.15, *n* = 9, *Dcn*^−/−^ = 1.83 ± 0.23, *n* = 10, *p* = 0.040) and *CTGF/CCN2* (WT = 1 ± 0.15, *n* = 7, *Dcn*^−/−^ = 1.84 ± 0.23, *n* = 7, *p* = 0.044) are significantly increased in the ON of DCN-deficient mice. * are significant with *p* ≤ 0.05. (**B**) Immunofluorescence staining against CTGF/CCN2 in the ON. Signal for CTGF/CCN2 is markedly stronger in the ON of the *Dcn*^−/−^ animal compared to its WT littermate. Blue: DAPI, red: CTGF/CCN2, ON = optic nerve, ONH = optic nerve head, Re = retina. Scale bars: 20 µm.

We previously reported that the absence of DCN leads to a glaucomatous phenotype in mice, with an increased expression of growth factors and ECM components in the region of the trabecular meshwork (TM) [32]. Analysis of mRNA levels of ONs obtained from DCN-deficient mice and their wildtype (WT) littermates revealed an upregulation of *TGF-β2* (WT = 1 ± 0.13, *n* = 9, *Dcn^−/−^* = 1.85 ± 0.23, *n* = 10, *p* = 0.036), *TGF-β1* (WT = 1 ± 0.15, *n* = 9, *Dcn^−/−^* = 1.83 ± 0.23, *n* = 10, *p* = 0.040) as well as *CTGF/CCN2* (WT = 1 ± 0.15, *n* = 7, *Dcn^−/−^* = 1.84 ± 0.23, *n* = 7, *p* = 0.044) (Figure 1A). Immunofluorescence staining against CTGF/CCN2 in sagittal sections through the ON underscored these findings, showing a substantially stronger signal for CTGF/CCN2 in DCN-deficient animals (Figure 1B).

### 2.2. Astrocytes of the ON and ONH Produce DCN

**Figure 2:**
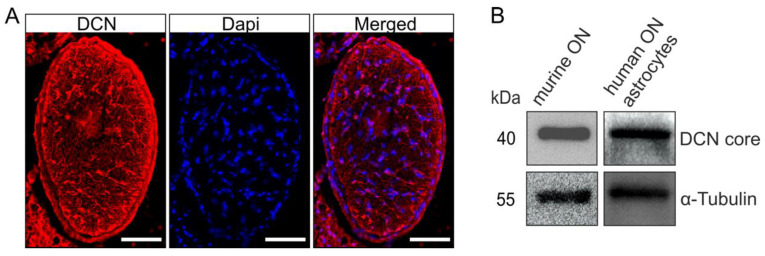
DCN is present in the human ONH and murine ON. A positive signal for DCN was observed in cross sections through murine ONs (**A**, scale bar 50 µm). Presence of DCN in the murine ON was confirmed via Western blot; Western blot analysis also showed that human ONH astrocytes produce DCN (**B**).

Immunofluorescence staining against DCN in cross sections through murine ONs revealed the presence of DCN (Figure 2A), with a unique distribution pattern, most prominent in distinct processes of the ON astrocytes [43]. The finding that astrocytes are the main source of DCN production in the ON was further corroborated by Western blot analysis using protein extracts from murine ONs. In the protein extracts of cultured human ONH astrocytes we also found a positive signal for DCN in the Western blot analysis (Figure 2B), showing that the DCN expression and synthesis by astrocytes is found throughout the species.

### 2.3. Reciprocal Negative Regulation of TGF-β and DCN in Human ONH Astrocytes and Murine ON Astrocytes

**Figure 3:**
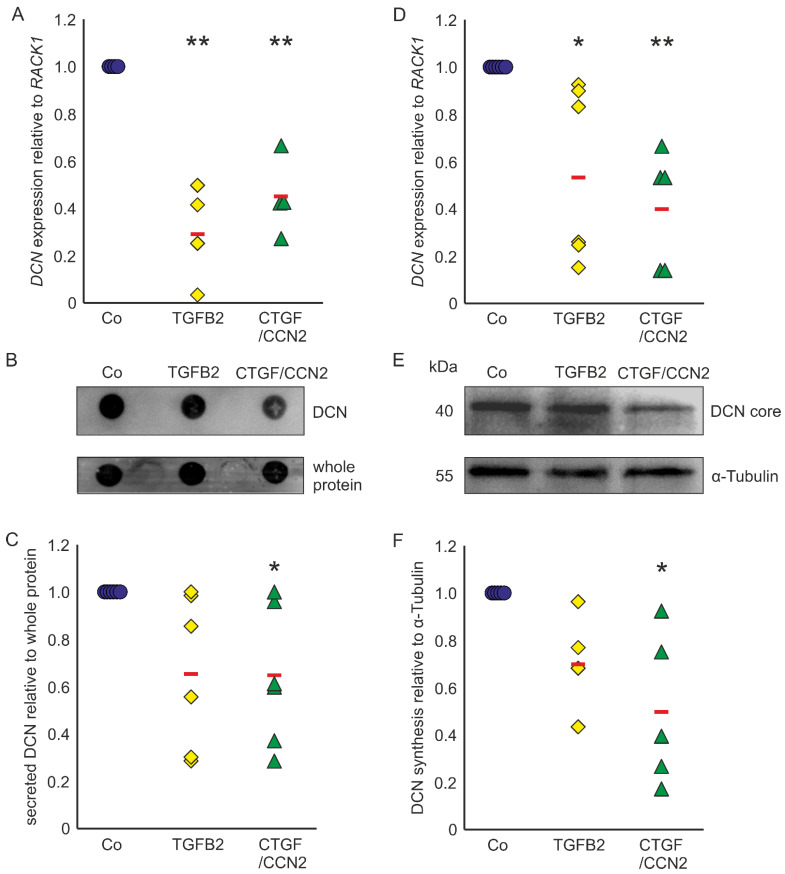
TGF-β and CTGF/CCN2 negatively regulate DCN in murine ON astrocytes (left) and human ONH astrocytes (right). (**A**) Treatment with TGF-β2 reduced *DCN* mRNA to 0.29 ± 0.22 (*n* = 4, *p* = 0.0003) and treatment with CTGF/CCN2 reduced *DCN* expression to 0.45 ± 0.16 (*n* = 4, *p* = 0.0019) in murine ON astrocytes. mRNA expression was normalized to *RACK1*. Expression in untreated controls was set to 1. (**A**) In the medium of murine ON astrocytes secreted DCN levels were at 0.66 ± 0.31 (*n* = 6, *p* = 0.089) after treatment with TGF-β2, while CTGF/CCN2 treatment significantly reduced DCN synthesis to 0.65 ± 0.26 (*n* = 6, *p* = 0.048). Secreted protein was normalized to whole protein, untreated controls were set to 1; (**B**,**C**). In human ONH astrocytes treatment with TGF-β2 reduced *DCN* mRNA to 0.56 ± 0.37 (*n* = 6, *p* = 0.0230) and treatment with CTGF/CCN2 reduced *DCN* expression to 0.40 ± 0.24 (*n* = 5, *p* = 0.0072). mRNA expression was normalized to *RACK1*. Expression in untreated controls was set to 1; (**D**) DCN protein levels were at 0.71 ± 0.11 (*n* = 4, *p* = 0.1861) after treatment with TGF-β2 (0.004 nM), while CTGF/CCN2 (3 nM) treatment significantly reduced DCN synthesis to 0.50 ± 0.18 (*n* = 5, *p* = 0.0129) in human ONH astrocytes (protein synthesis was normalized to α-Tubulin, expression in untreated control cells was set to 1); (**E**,**F**). Co = Control. *, ** are significant with *p* ≤ 0.05 or *p* ≤ 0.01.

Astrocytes of the ONH react to TGF-β treatments with upregulation of ECM expression [29,30,31] and show reactivity in POAG [17,18,43]. In murine ON astrocytes *DCN* expression was reduced to 0.29 ± 0.22 (*n* = 4, *p* = 0.0003) after treatment with TGF-β2 and to 0.45 ± 0.16 (*n* = 4, *p* = 0.0019) after treatment with CTGF/CCN2 (Figure 3A). Amounts of secreted DCN of murine ON astrocytes were significantly lowered by treatments with CTGF/CCN2 (0.65 ± 0.26; *n* = 6, *p* = 0.048). After TGF-β2 treatment the DCN synthesis was at 0.66 ± 0.31 (*n* = 6, *p* = 0.089) in the murine ON astrocytes (Figure 3B,C). Analysis of *DCN* expression in human ONH astrocytes showed that compared to untreated controls, treatment with TGFβ-2 led to a down regulation of *DCN* mRNA to 0.56 ± 0.37 (*n* = 6, *p* = 0.0230) and treatment with CTGF/CCN2 reduced *DCN* expression to 0.40 ± 0.24 (*n* = 5, *p* = 0.0072, Figure 3D). DCN synthesis was significantly reduced by CTGF/CCN2 treatment (0.50 ± 0.37; *n* = 5, *p* = 0.013), whereas the DCN protein level was at 0.71 ± 0.22 (*n* = 4, *p* = 0.186) after TGF-β2 treatment (Figure 3E,F). In human ONH astrocytes and in murine ON astrocytes CTGF/CCN2 treatment had a stronger negative regulatory effect on *DCN* expression than TGF-β2 treatment.

**Figure 4:**
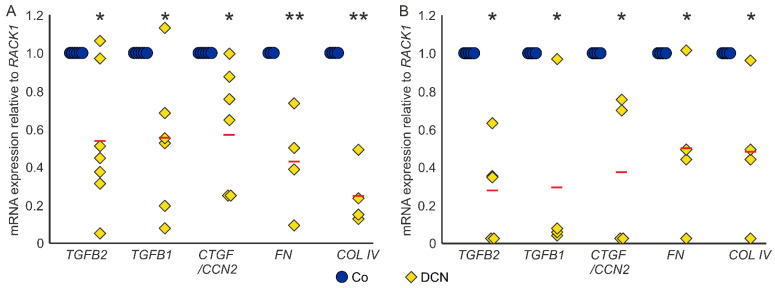
DCN reduces expression of TGF-βs and response genes in murine ON astrocytes (**A**) and human ONH astrocytes (**B**). (**A**) Expression levels of *TGF-βs, CTGF/CCN2, FN* and *COL IV* were reduced in murine ON astrocytes after treatment with 25 nM DCN. *TGF-β2*: 0.54 ± 0.34, *n* = 7, *p* = 0.006, *TGF-β1*: 0.56 ± 0.38, *n* = 6, *p* = 0.026, CTGF/CCN2: 0.59 ± 0.30, *n* = 7, *p* = 0.005, *FN*: 0.43 ± 0.21, *n* = 4, *p* = 0.005 and *COL IV*: 0.25 ± 0.13, *n* = 4, *p* = 0.0001. mRNA expression was normalized to *RACK1*. Expression in untreated controls was set to 1. (**B**) Expression levels of *TGF-βs*, *CTGF/CCN2, FN* and *COL IV* were reduced in human ONH astrocytes after treatment with 25 nM DCN. *TGF-β2:* 0.27 ± 0.24, *n* = 5, *p* = 0.015; *TGF-β1*: 0.29 ± 0.40, *n* = 4, *p* = 0.02; *CTGF/CCN2*: 0.37 ± 0.36, *n* = 4, *p* = 0.011; *FN*: 0.49 ± 0.25, *n* = 4, *p* = 0.049; *COL IV*: 0.47 ± 0.17, *n* = 4, *p* = 0.032. mRNA expression was normalized to *RACK1*. Expression in untreated controls was set to 1. *, ** are significant with *p* ≤ 0.05 or *p* ≤ 0.01.

Conversely, treatment with DCN led to a significant downregulation of *TGF-β2* (0.54 ± 0.34, *n* = 7, *p* = 0.006), *TGF-β1* (0.56 ± 0.38, *n* = 6, *p* = 0.026) and *CTGF/CCN2* (0.59 ± 0.30, *n* = 7, *p* = 0.005) in murine ON astrocytes compared to untreated controls. Furthermore, DCN treatment significantly lowered the mRNA expression of the ECM components *FN* (0.43 ± 0.21, *n* = 4, *p* = 0.005) and *COL IV* (0.25 ± 0.13, *n* = 4, *p* = 0.0001), which are target genes of TGF-βs and CTGF/CCN2 (Figure 4A). In coherence, treatment with DCN reduced expression of all five genes in human ONH astrocytes (*TGF-β2:* 0.27 ± 0.24, *n* = 5, *p* = 0.015; *TGF-β1*: 0.29 ± 0.40, *n* = 4, *p* = 0.02; *CTGF/CCN2*: 0.37 ± 0.36, *n* = 4, *p* = 0.011; *FN*: 0.49 ± 0.25, *n* = 4, *p* = 0.049; *COL IV:* 0.47 ± 0.17, *n* = 4, *p* = 0.032, Figure 4B).

### 2.4. DCN Suppresses TGF-β and CTGF/CCN2 Expression via the pAKT/AKT Signaling Pathway

**Figure 5:**
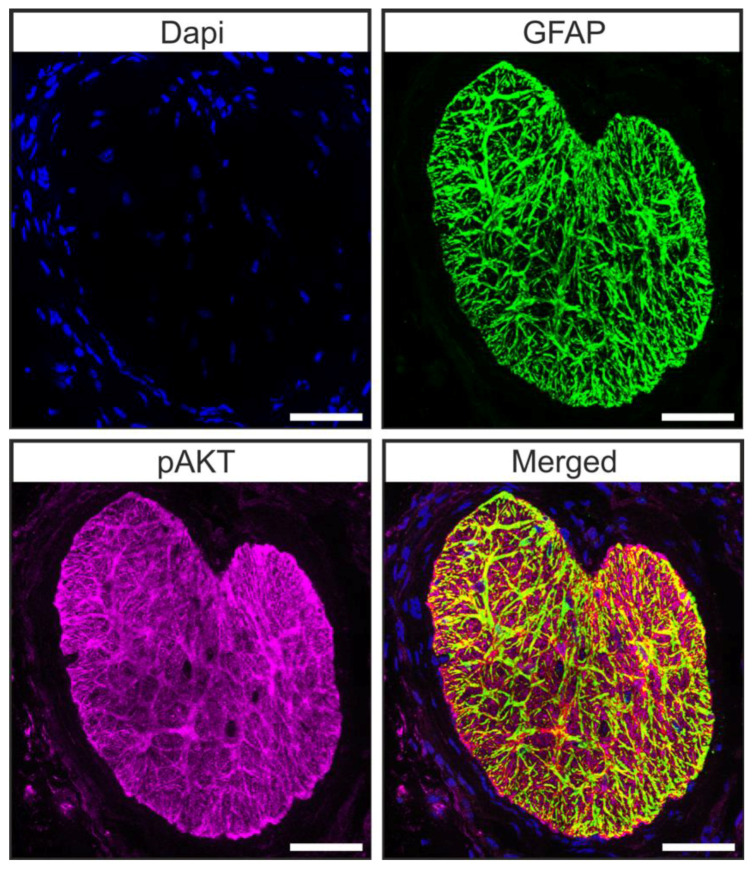
Astrocytes of the ON engage in pAKT/AKT signaling. Double staining against GFAP (green) and pAKT (magenta) revealed that pAKT/AKT signaling is active in astrocytes of the murine ON. Scale bars 50 µm [45].

Microarray analysis of the transcriptome of brain astrocytes showed that the pAKT/AKT signaling pathway is active in these cells [44]. Hence, we carried out double staining against pAKT and glial fibrillary acidic protein (GFAP) in tangential sections of the glial lamina of 12-week-old animals to investigate whether astrocytes of the murine glial lamina engage in pAKT/AKT signaling. We found a co-localization of pAKT and GFAP in glial lamina (Figure 5) [45], showing that pAKT/AKT signaling is active in astrocytes of this region.

**Figure 6:**
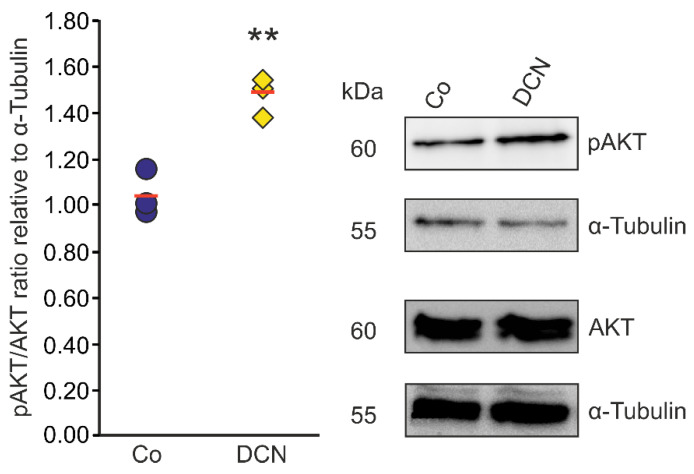
DCN activates AKT signaling in murine ON astrocytes. pAKT/AKT ratio was significantly higher in murine ON astrocytes treated with 25 nM of DCN for 6 h (1.47 ± 0.09, *n* = 3, *p* ≤ 0.01) in comparison to untreated controls (1.04 ± 0.09, *n* = 3) speaking for an activation of the pAKT/AKT signaling pathway in this cell type. pAKT and AKT were normalized to α-Tubulin. ** are significant with *p* ≤ 0.01.

Since there is evidence in other cell types that DCN negatively regulates TGF-β via the pAKT/AKT signaling pathway [46] we aimed to analyze if the regulation of *TGF-β* and *CTGF/CCN2* by DCN in murine ON astrocytes is also mediated via this pathway. Accordingly, we treated murine ON astrocytes with 25 nM DCN for 6 h to analyze its possible ability to activate the pAKT/AKT signaling pathway. We found an increase of pAKT in relation to AKT (Figure 6). The pAKT/AKT ratio of treated cells was 1.47 ± 0.09 (*n* = 3, *p* = 0.004) compared to untreated control cells (1.04 ± 0.09, *n* = 3); thus, we conclude that DCN treatment leads to an activation of the pAKT/AKT pathway in murine ON astrocytes.

**Figure 7:**
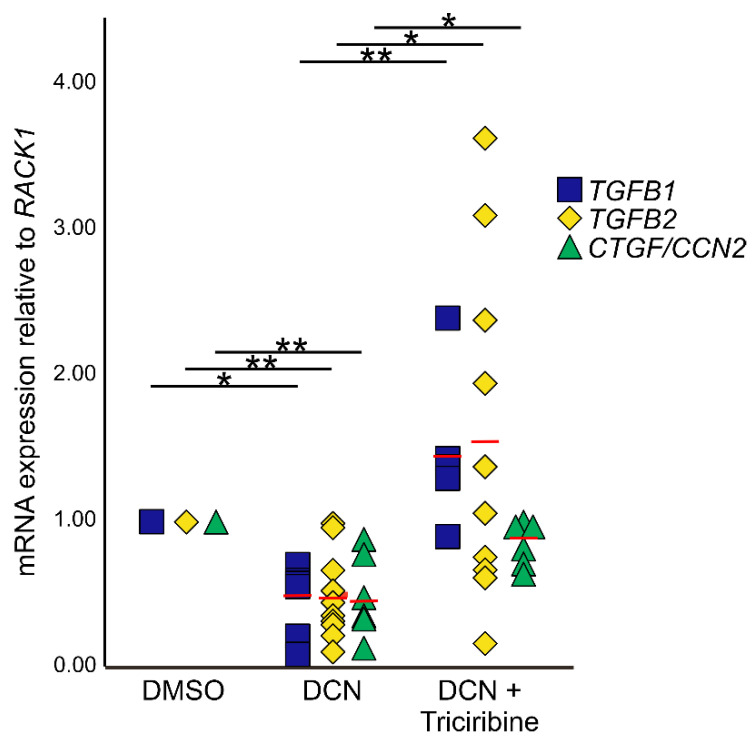
Inhibition of the pAKT/AKT signaling pathway circumvents negative regulation of TGF-β and CTGF/CCN2 by DCN. After treatment with DCN expression of *TGF-β1* (0.44 ± 0.10, *n* = 6, *p* ≤ 0.05); *TGF-β2* (0.48 ± 0.09, *n* = 10, *p* ≤ 0.001) and *CTGF/CCN2* (0.49 ± 0.12, *n* = 6, *p* ≤ 0.001) was significantly lower than in DMSO treated controls (n*_TGF-β1_* = 6, n*_TGF-β2_* = 10, n*_CTGF_* = 6). Combined treatment with triciribine and DCN did not lead to changes in expression compared to untreated controls, but the downregulation was significantly inhibited compared to cells treated with DCN only (*TGF-β1*: 1.46 ± 0.24, *n* = 5, *p* ≤ 0.001; *TGF-β2*: 1.56 ± 0.36, *n* = 10, *p* ≤ 0.05; *CTGF/CCN2*: 0.84 ± 0.06, *n* = 6, *p* ≤ 0.05). Expression was normalized to *RACK1*. *, ** are significant with *p* ≤ 0.05 or *p* ≤ 0.01.

To analyze if the negative effects of DCN on expression of *TGF-β1, 2* and *CTGF/CCN2* are mediated via pAKT/AKT signaling, murine astrocytes were treated with the AKT signaling inhibitor triciribine in combination with DCN. Treatment with DCN resulted in a significant downregulation of all three growth factors (Figure 7; *TGF-β2*: 0.48 ± 0.09, *n* = 10, *p* = 0.001; *TGF-β1*: 0.44 ± 0.10, *n* = 6, *p* = 0.03; *CTGF/CCN2*: 0.49 ± 0.12, *n* = 6, *p* = 0.0006). After combined treatment with triciribine and DCN, triciribine attenuated effects of DCN on *CTGF/CCN2, TGF-β1*, and *TGF-β2* mRNA expression (Figure 7; *TGF-β2*: 1.56 ± 0.36, *n* = 10, *p* = 0.031; *TGF-β1*: 1.46 ± 0.24, *n* = 5, *p* = 0.0004; *CTGF/CCN2*: 0.84 ± 0.06, *n* = 6, *p* = 0.013), whereas combined treatment with triciribine and DCN did not lead to changes in expression compared to untreated controls (*p_TGF-β1_* = 0.85; *p_TGF-β2_* = 0.32; *p_CTGF_* = 0.29).

## 3. Discussion

We conclude that DCN is an important regulator of *TGF-β1* and *2* as well as *CTGF/CCN2* in astrocytes of the ON and ONH and that DCN administrates the negative effect on the expression of both growth factors via the pAKT/AKT signaling pathway. Vice versa, TGF-β and CTGF/CCN2 reduce the expression and synthesis of DCN in ON astrocytes. In DCN-deficient mice the profibrotic growth factors TGF-β1, TGF-β2 and CTGF/CCN2, which are involved in the structural remodeling of the ONH, are upregulated in this region. The increased amounts of these growth factors would result in a vicious circle by the downregulation of the endogenous inhibitor DCN, resulting in a fortified remodeling of the ONH in glaucoma. This conclusion rests on (1) the elevated expression of *TGF-β1*, *TGF-β2* and *CTGF/CCN2* in DCN-deficient mice; (2) the suppression of *DCN* expression in cultured ON and ONH astrocytes after CTGF/CCN2 and TGF-β2 treatment and reduced synthesis after CTGF/CCN2 treatment; (3) the finding that DCN attenuates the expression of *TGF-β* and of typical target genes of the TGF-β signaling pathway in cultured ON astrocytes; (4) the fact that combined treatment with DCN and triciribine, a small molecule inhibitor of Akt signaling, attenuated effects of DCN on *CTGF/CCN2*, *TGF-β1*, and *TGF-β2* mRNA expression.

The remodeling processes in the ONH of glaucomatous patients is accompanied by a disruption of the homeostatic balance of growth factors. TGF-β2 was one of the first growth factors identified in higher amounts in the ONH of glaucoma patients [28,29]. An IOP-dependent mechanism is suggested, as TGF-β was also elevated in the glaucomatous monkey ONH [47,48] and as in murine glaucoma models the TGF-β signaling pathway was activated in the ONH [49]. A correlation of TGF-β expression/signaling and biomechanical strain was proven in cell culture studies of ONH astrocytes [50] and lamina cribrosa cells [51]. Along with the increase of TGF-β expression in glaucomatous lamina cribrosa cells, a significant downregulation of *DCN* was observed [51] which was coherent with expression data from glaucomatous Schlemm canal endothelial cells [42]. In the ON of DCN-deficient mice, a dramatic upregulation of *CTGF/CCN2*, *TGF-β-1* and *-2* was found, whereas we could show for the first time that DCN is highly expressed in the ON of wildtype animals. The in vitro studies of the ON and ONH astrocytes, derived from mice and humans, showed that astrocytes could be the source of the DCN signal throughout the species. The function of DCN in this region is yet unknown, but our data strongly hint towards a negative regulatory mechanism of the TGF-β and CTGF/CCN2 pathways.

The observed reciprocal effects of TGF-β2, CTGF/CCN2 and DCN have not been studied up till now in human ONH astrocytes or murine ON astrocytes in vitro. In other cell types the influence of TGF-β on DCN levels was investigated and results indicate that it is cell type- or tissue-specific. TGF-β treatment leads to a decreased *DCN* expression in human skin fibroblasts and human chondrocytes [52,53] while *DCN* expression is upregulated after TGF-β treatment in murine osteoblasts and rat mesangial cells [54]. Recently we could demonstrate that TGF-β2 and DCN as well as CTGF/CCN2 and DCN have negative reciprocal effects on mRNA expression and protein synthesis in the TM in vitro and in vivo [32]. In murine ON astrocytes and human ONH astrocytes, treatment with CTGF/CCN2 and TGF-β2 caused a significant reduction in *DCN* mRNA expression, whereas only CTGF/CCN2 led to a significant reduction of DCN synthesis in both cell lines. The TGF-β2 treatment showed no significant reduction of the DCN levels, which might be due to the time course. In the future, long term TGF-β2 treatments should be analyzed. The negative reciprocal effects appear to be the most common mechanism in ocular cells and tissues affected during the pathological changes in glaucoma. A disturbance of the regulatory system would most likely lead to a self-enhancing positive feedback loop.

Along with the increase of TGF-β2, a substantial alteration of the ECM occurs in the ONH and in the peripapillary sclera of glaucomatous patients. Astrocytes play a pivotal role in the restructuring process by de novo collagen synthesis [55], which occurs in an IOP dependent manner [56]. A lack of DCN in the ON and ONH could contribute to the observed disorganization of ECM in ONH and the peripapillary sclera in glaucomatous eyes [57,58,59], since DCN is essential for binding and arranging collagen fibers [60,61]. Furthermore, TGF-β2 treatment causes an increase in ECM expression in ONH astrocytes [29,30,31] and this upregulation is mediated via CTGF/CCN2 [30]. By reducing the expression and synthesis of TGF-β and CTGF/CCN2, DCN should be able to lower their profibrotic effects. We could give first proof of this hypothesis by showing reduced expression of *FN* and *COL IVa2* of ON astrocytes after treatment with DCN. 

Alterations in the ECM arrangement of the lamina cribrosa and the peripapillary sclera cause changes in the mechanical support of this region and thereby affect the mechanosensation mechanisms of astrocytes by their junctional complexes upon IOP elevation [55]. In DCN-deficient mice we reported previously that the increase in IOP and the loss of ON axons is accompanied by an enhanced expression and synthesis of GFAP by astrocytes [32], which, from a biomechanical perspective, would cause together with ECM changes an increase in tissue stiffness [62,63]. Up to now, there has been no data on the amounts of DCN in the ONH of glaucoma patients, which would be of great interest since increased amounts of TGF-β are known to be present in the ONH [26,28,47]

DCN can influence the activity and expression of TGF-β via different pathways, firstly via direct binding [64] and secondly via the upregulation of fibrillin-1 (FBN1) synthesis. While a negative influence of DCN on the biological activity of CTGF/CCN2 has been proven [38], there is no data on the influence of DCN on the expression and synthesis of this growth factor in the ON or ONH.

We demonstrate that the pAKT/AKT signaling pathway is active in murine astrocytes of the ON. Up until now activity of this pathway has been shown in human brain-derived astrocytes [65] and the effect of DCN on this pathway in astrocytes has not been studied before. Activation of the pAKT/AKT pathway by DCN leads to an increased synthesis of FBN1 in rat kidney fibroblasts [66] and FBN1 can actively interfere with TGF-β by preventing the release of active TGF-β from the latent TGF-β complex [46,67]. In glaucomatous lamina cribrosa cells a significant downregulation of FBN-1 and DCN, together with an increased expression of TGF- β and thrombospondin-1, an activator of TGF-β [68], was described [51]. Mutation of FBN1 causes Marfan syndrome [69,70] and about 2% of Marfan patients are affected by a not-categorized type of glaucoma [71]. This hints that the mutation of FBN1 leads to hyperactivity of TGF-β and could thereby favor the onset of glaucoma. By inhibiting the pAKT/AKT signaling pathway, we demonstrated that DCN regulates *TGF-β1*, *TGF-β2* and *CTGF/CCN2* expression via this pathway in murine ON astrocytes. However, our study does not completely answer the question of whether the effect on *CTGF/CCN2* is direct or mediated by the down-regulation of *TGF-β* or an altered activity of TGF-β due to changes in the synthesis of FBN1.This is an important question to address in future experiments. Since we could observe an inhibitory effect on DCN’s regulation of *TGF-β* and *CTGF/CCN2* by triciribine after 24 h, it is very likely that DCN directly regulates them via AKT signaling. Furthermore, there is evidence that pAKT can bin to Smad3 and inhibit its phosphorylation, resulting in an inhibition of TGF-β signaling [72].

This study is clearly limited by having shown the reciprocal effects and pathway analysis in vitro only. Further, we are aware that our study does not exclude the possibility that DCN regulates TGF-βs and CTGF via direct binding in our experimental design. 

Nevertheless, we strongly believe that all these results make DCN an interesting candidate for future treatments of glaucoma due to its ability to reduce expression and synthesis as well as activity of TGF-β and CTGF/CCN2 in human and murine astrocytes of the ON and ONH.

## 4. Materials and Methods

### 4.1. Animals

For in vivo experiments *Dcn^−/−^* in a 129/Sv—C57BL/6—CD1 mixed background have been used. *Dcn^−/−^* mice were generated and characterized before. DCN-deficient mice were generated via the insertion of a Pgk-Neomycin cassette into exon 2 of the *Dcn* gene, resulting in a complete absence of *DCN* on the mRNA and protein level [73]. Heterozygous *Dcn^+/−^* animals were mated, resulting in homozygous knockout, homozygous wildtype (WT) and heterozygous offspring. For data analysis, only homozygous WT and homozygous DCN-deficient mice were analyzed. For further experiments, CD1 WT mice were used. Mice were housed under standardized conditions of 62% air humidity and 21 °C room temperature. Feeding was ad libitum. Animals were kept at a 12 h light/ dark cycle (6 a.m. to 6 p.m.). All experiments conformed to the tenets of the National Institutes of Health Guidelines on the Care and Use of Animals in Research, the EU Directive 2010/63/E and the Association for Research in Vision and Ophthalmology Statement for the Use of Animals in Ophthalmic in Vision Research, and were approved by local authorities (54-2532.1-44/12; Regierung Oberpfalz, Bavaria, Germany).

### 4.2. Cell Culture

Three-week old CD1 mice of mixed sex were used for the isolation of ON astrocytes. After mice were sacrificed, both eyes were enucleated, and the ONs were cut off the globe. After removal of the dura, ON samples were digested in 200 µL Trypsin (Gibco BRL, Karlsruhe, Germany) for 30 min at 37 °C. The tissue was then sheared by repeated pipetting and plated on laminin-coated 6-well plates. Cells were grown in DMEM/F12 (Gibco BRL, Karlsruhe, Germany) enriched with 10% fetal bovine serum (FBS, Gibco BRL, Karlsruhe, Germany), 1% penicillin/streptomycin (Gibco BRL, Karlsruhe, Germany) and 1% astrocyte growth supplement (Sciencell, Carlsbad, CA, USA). Medium was not changed in the following seven days to allow the cells to attach to the tissue culture plates. After seven days, the medium was replaced two times a week with fresh medium. A pure astrocyte culture was obtained by shaking the wells for 12 h to remove less-adhesive cells. Cells were maintained in an incubator at 37 °C and 5% CO2. After cells grew to confluence, they were seeded in 25 cm^2^ cell culture flasks (Nunc, VWR, Darmstadt, Germany). Astrocytes were characterized via GFAP staining. Only cells from passage 2 to 10 were used for experiments.

Cultures of human ONH astrocytes were established from the eyes of human donors according to protocols published previously [30]. The age of the donors ranged from 34 to 76 years. Human ONH astrocytes of the third to fifth passage were seeded in 35-mm culture wells (4.0 × 10^5^ cells/well) and grown to a confluent monolayer in DMEM F12 medium plus 10% (*v*/*v*) fetal bovine serum, 100 U/mL penicillin, 100 μg/mL streptomycin in 7% CO2 at 37 °C (PAA, Pasching, Austria). Methods for securing human tissues were humane, included proper consent and approval, and complied with the Declaration of Helsinki.

The confluent murine or human cells were incubated in serum-free medium for 24 h followed by incubation in fresh serum-free medium. To analyze effects of TGFβ-2, CTGF/CCN2 and DCN, astrocytes were treated with 4 pM TGF-β2 (R&D Systems, Minneapolis, Minnesota, USA), 3 nM CTGF/CCN2 (Prospec, Rehovot, Israel) or 25 nM DCN (R&D Systems, Minneapolis, MI, USA) for 24 h. Untreated cells served as controls. To investigate the effect of DCN on the AKT-signaling pathway, murine astrocytes were treated with 25 nM DCN for 6 h. To reassess if DCN regulates expression of TGF-β1, TGF-β2 and CTGF/CCN2 via the the AKT signaling pathway in murine astrocytes, cells were treated with 25 nM DCN only or in combination with the AKT signaling inhibitor Triciribine (Selleckchem, Houston, TX, USA). Cells treated with DMSO (Roth, Karlsruhe, Germany) served as control, since Triciribine was solubilized in DMSO.

### 4.3. RNA Analysis

Total RNA of cells was extracted with peqGold TrifastTM (VWR, Darmstadt, Germany) according to manufacturer’s recommendations. First strand cDNA was prepared from total RNA using the qScript™ cDNA Synthesis Kit (Quanta, Gaithersburg, MD, USA) according to the manufacturer’s instructions. Real-time reverse transcription polymerase chain reaction (RT-PCR) was performed on a BioRad iQ5 Real-time PCR Detection System (Bio-Rad, Hercules, CA, USA) using the following temperature profile: 40 cycles of 10 s melting at 95 °C, 40 s of annealing and extension at 60 °C. Primer pairs (Appendix A, Table A1) were purchased from Invitrogen and extended over exon–intron boundaries. RNA that was not reversely transcribed served as the negative control for real-time RT-PCR. Receptor of activated protein C kinase 1 (*RACK1*) was used as housekeeping genes for relative quantification of the real-time RT-PCR experiments. To allow for relative quantification, we identified housekeeping genes by using Genex software version 5.3.2 (MultiD Analysis, Göteburg, Sweden) [74]. In initial experiments, real-time RT-PCR for the potential housekeeping genes *RACK1*, *GAPDH*, *RPL32*, *β-tubulin* and *RPS9* were performed for mouse tissue and for each of the treatment protocols. C_T_ values were loaded to the software, which distinguishes genes that are regulated in a specific condition from those that are likely not. Best results were obtained for *RACK1.* Quantification was performed with iQ5 Standard-Edition (Version 2.0.148.60623) software (Bio-Rad, Hercules, CA, USA).

### 4.4. Western Blot Analysis

Protein extracts of cells were extracted with peqGold TrifastTM (VWR, Radnor, PA, USA) according to manufacturer’s recommendations, and protein content was measured with the bicinchoninic acid protein assay (ThermoFisher Scientific, Waltham, MA, USA). Alternatively, cell culture medium was collected and used directly for Western blotting. Proteins were separated by SDS-PAGE and transferred to polyvinylidene fluoride membranes. Western blot analysis was performed with specific antibodies as described previously [75]. Antibodies were used as follows: rabbit anti-human/mouse-DCNH80 (1:200; Santa Cruz, CA, USA), rabbit anti-AKT (1:1000; cell signaling, Cambridge, England), rabbit anti-pAKT (1:1000; cell signaling, Cambridge, England), donkey anti-rabbit-horseradish peroxidase (HRP) and chicken anti-rabbit-AP (1:2000; all Santa Cruz, CA, USA). Chemiluminescence was detected on a LAS 3000 imaging workstation (Raytest, Straubenhardt, Germany). α -tubulin (rabbit anti-α-tubulin, 1:2500, Rockland Immunochemicals Inc., Gilbertsville, PA, USA) was used as loading control to normalize the signal intensity of the Western blots. The intensity of the bands detected by Western blot analysis was determined using appropriate software (AIDA Image analyzer software, Raytest, Straubenhardt, Germany).

### 4.5. Dot Blot Analysis

To analyze levels of secreted DCN in the media of murine astrocytes after treatments with TGFβ-2 or CTGF, medium was taken off the cells and stored at −80 °C till further use. Secreted protein was blotted on PVDF membranes via dot blotting. Initially, the membrane was activated in methanol, then subsequently equilibrated in transfer buffer. One layer of Whatman paper, soaked in transfer buffer, was placed on the lower part of the Dot Blotter (Schleicher & Schuell, Dassel, Germany). The PVDF membrane was placed on top of the Whatman paper and the upper part of the Dot Blotter was placed onto the membrane. The Dot Blotter was connected to an aspirator hose to aspirate the medium through the membrane. 100 μL of medium were loaded per dot. After the medium was aspirated completely, the membrane was washed in TBST shortly and then incubated in 5% BSA for 1 h at RT. Incubation with antibodies and detection of chemiluminescence signal were carried out as described in Section 4.4.

### 4.6. Immunofluorescence

Eyes were obtained from CD1 wildtype mice at 3 months of age. Eyes were enucleated and fixed in 4% (*w*/*v*) PFA in phosphate-buffered saline (PBS) for 1 h. After fixation, eyes were equilibrated in 10%, 20%, and 30% sucrose for 4 h, embedded in Tissue-Tek optimal cooling temperature compound (Sakura Finetek Europe B.V., Zoeterwoude, The Netherlands), and stored at −20 °C. Frozen sections were cut on Microm HM500 OM Cryostat (Microm International, Walldorf, Germany). After blocking with 2% bovine serum, 0.2% cold water fish skin gelatin (Sigma-Aldrich, St. Louis, MO, USA), and 0.1% Triton-X-100 in 0.1 M phosphate buffer for 1 h at room temperature, frozen sections were incubated with chicken anti-GFAP (1:1000; LS Bio, Seattle, DC, USA) and/or rabbit anti-pAKT (1:500) at 4 °C overnight, or with rabbit anti-DCN (1:500, LF113, donated by L. Fisher). Afterwards, tissue sections were washed three times with 0.1 M phosphate buffer followed by incubation for 1 h at room temperature with Cy3™ goat anti-rabbit (1:2000, Jackson Immuno Research Europe Ltd., Suffolk, UK) and/or Alexa Flour 488 goat anti-chicken (1:1000, ThermoFisher Scientific, Waltham, Massachusetts, USA). As a control for unspecific binding of secondary antibodies, negative controls were performed, which were handled similarly but incubated in PBS without primary antibodies. After washing three times with PBS, the slides were mounted using the DakoCytomation fluorescent mounting medium with DAPI 1:10 (Agilent, Santa Clara, CA, USA). Slides were dried overnight at 4 °C before microscopy. Immunofluorescence was visualized using a Zeiss Axio Imager fluorescence microscope (Carl Zeiss AG). Images were taken using the same exposure times.

### 4.7. Number of Experiments and Statistical Analysis

To assess the effects of treatments, each Western blot experiment was repeated at least three times with protein extract or culture media from primary human ONH astrocytes or murine ON astrocytes of different donors/mice, respectively. Each real-time RT-PCR analysis was performed in duplicate and repeated at least three times. All data is presented as mean ± SEM. Normal distribution of data was ensured using the Kolmogorov–Smirnov Test with Lilliefors correction. Student’s t-test was used for statistical analysis of the protein and RNA data of cells treated with DCN only. Data CTGF and TGF-β2 treatments as well as data of DCN and triciribine co-treatments were analyzed using one-way ANOVA with Tukey’s multiple comparisons test.

## Appendix A

**Table A1:** Sequences and positions of primer pairs used for real-time RT-PCR.

Primer	Species	Orientation	Sequence 5′ to 3′	Position
*COL IV a2*	*Homo sapiens*	forward reverse	acaggacagaaaggagacca ggtgtgatgcctgggaac	4019–4038 4104–4087
*CTGF*	*Homo sapiens*	forward reverse	ctcctgcaggctagagaagc gatgcactttttgcccttctt	878–897 971–951
*DCN*	*Homo sapiens*	forward reverse	tcgagtggtccagtgttctg cctttttggtgttgtgtcca	280–299 384–365
*FN*	*Homo sapiens*	forward reverse	ccctgattggaaggaaaaaga atgaagattggggtgtggaa	6217–6237 6284–6265
*RACK1*	*Homo sapiens*	forward reverse	ctagaatgatctttccctctaaatcc cctaaccgctactggctgtg	170–188 241–222
*TGF-β 1*	*Homo sapiens*	forward reverse	gcagcacgtggagctgta cagccggttgctgaggta	1373–1390 1436–1419
*TGF-β 2*	*Homo sapiens*	forward reverse	ccaaagggtacaatgccaac cagatgcttctggatttatggtatt	2379–2398 2492–2468
*COL IV a2*	*Mus musculus*	forward reverse	ctgggttcccaggattca agagtctcctttattcctttgg	1917–1934 1990–1968
*CTGF*	*Mus musculus*	forward reverse	tgacctggaggaaaacattaaga agccctgtatgtcttcacactg	1013–1035 1124–1103
*DCN*	*Mus musculus*	forward reverse	gagggaactccacttggaca ttgttgttgtgaaggtagacgac	1039–1058 1112–1134
*RACK1*	*Mus musculus*	forward reverse	tctgcaagtacacggtccag gagacgatgatagggttgctg	514–533 604–584
*FN*	*Mus musculus*	forward reverse	cggagagagtgcccctacta cgatattggtgaatcgcaga	4327–436 4403–4384
*TGF-β 1*	*Mus musculus*	forward reverse	tggagcaacatgtggaactc gtcagcagccggttacca	1358–1377 1430–1413
*TGF-β 2*	*Mus musculus*	forward reverse	tcttccgcttgcaaaacc gtgggagatgttaagtctttgga	5361–5378 5451–5429

## References

[1] Quigley H.A., Broman A.T. (2006). The number of people with glaucoma worldwide in 2010 and 2020. Br. J. Ophthalmol..

[2] Resnikoff S., Pascolini D., Etya’Ale D., Kocur I., Pararajasegaram R., Pokharel G.P., Mariotti S.P. (2004). Global data on visual impairment in the year 2002. Bull. World Health Organ..

[3] Gharahkhani P., Jorgenson E., Hysi P., Khawaja A.P., Pendergrass S., Han X., Ong J.S., Hewitt A.W., Segrè A.V., Rouhana J.M. (2021). Genome-wide meta-analysis identifies 127 open-angle glaucoma loci with consistent effect across ancestries. Nat. Commun..

[4] Choquet H., Paylakhi S., Kneeland S.C., Thai K.K., Hoffmann T.J., Yin J., Kvale M.N., Banda Y., Tolman N.G., Williams P.A. (2018). A multiethnic genome-wide association study of primary open-angle glaucoma identifies novel risk loci. Nat. Commun..

[5] Gordon M.O., Beiser J.A., Brandt J.D., Heuer D.K., Higginbotham M.D., Johnson C.A., Keltner J.L., Miller J.P., Parrish R.K. (2002). Ocular Hypertension Treatment Study Group; et al. The Ocular Hypertension Treatment Study: Baseline factors that predict the onset of primary open-angle glaucoma. Arch. Ophthalmol..

[6] The AGIS Investigators (2000). The advanced glaucoma intervention study (AGIS): 7. The relationship between control of intraocular pressure and visual field deterioration. Am. J. Ophthalmol..

[7] Collaborative Normal-Tension Glaucoma Study Group (1998). Comparison of glaucomatous progression between untreated patients with normal-tension glaucoma and patients with therapeutically reduced intraocular pressures. Am. J. Ophthalmol..

[8] Collaborative Normal-Tension Glaucoma Study Group (1998). The effectiveness of intraocular pressure reduction in the treatment of normal-tension glaucoma. Am. J. Ophthalmol..

[9] Leske M.C., Heijl A., Hussein M., Bengtsson B., Hyman L., Komaroff E., Early Manifest Glaucoma Trial Group (2003). Factors for glaucoma progression and the effect of treatment: The early manifest glaucoma trial. Arch. Ophthalmol..

[10] Quigley H.A. (2015). The contribution of the sclera and lamina cribrosa to the pathogenesis of glaucoma: Diagnostic and treatment implications. Prog. Brain Res..

[11] Ransom B., Behar T., Nedergaard M. (2003). New roles for astrocytes (stars at last). Trends Neurosci..

[12] Hernandez M.R. (1992). Ultrastructural immunocytochemical analysis of elastin in the human lamina cribrosa. Changes in elastic fibers in primary open-angle glaucoma. Investig. Ophthalmol. Vis. Sci..

[13] Hernandez M.R., Agapova O.A., Yang P., Salvador-Silva M., Ricard C.S., Aoi S. (2002). Differential gene expression in astrocytes from human normal and glaucomatous optic nerve head analyzed by cDNA microarray. Glia.

[14] Hernandez M., Ye H., Roy S. (1994). Collagen Type IV Gene Expression in Human Optic Nerve Heads with Primary Open Angle Glaucoma. Exp. Eye Res..

[15] Schwab J.M., Postler E., Nguyen T.D., Mittelbronn M., Meyermann R., Schluesener H.J. (2000). Connective tissue growth factor is expressed by a subset of reactive astrocytes in human cerebral infarction. Neuropathol. Appl. Neurobiol..

[16] Stöckli K.A., Lottspeich F., Sendtner M., Masiakowski P., Carroll P., Götz R., Lindholm D., Thoenen H. (1989). Molecular cloning, expression and regional distribution of rat ciliary neurotrophic factor. Nat. Cell Biol..

[17] Varela H.J., Hernandez M.R. (1997). Astrocyte responses in human optic nerve head with primary open-angle glaucoma. J. Glaucoma.

[18] Hernandez M.R., Pena J.D. (1997). The optic nerve head in glaucomatous optic neuropathy. Arch. Ophthalmol..

[19] Netland P.A., Ye H., Streeten B.W., Hernandez M.R. (1995). Elastosis of the lamina cribrosa in pseudoexfoliation syndrome with glaucoma. Ophthalmology.

[20] Pena J.D., Netland P.A., Vidal I., Dorr D.A., Rasky A., Hernandez M. (1998). Elastosis of the Lamina Cribrosa in Glaucomatous Optic Neuropathy. Exp. Eye Res..

[21] Quigley H.A., Brown A., Dorman-Pease M.E. (1991). Alterations in elastin of the optic nerve head in human and experimental glaucoma. Br. J. Ophthalmol..

[22] Quigley H.A., Pease M., Brown A.E. (1991). Quantitative study of collagen and elastin of the optic nerve head and sclera in human and experimental monkey glaucoma. Curr. Eye Res..

[23] Hernandez M.R., Wang N., Hanley N.M., Neufeld A.H., Hernandez M.R., Wang N., Hanley N.M., Neufeld A.H. (1991). Localization of collagen types I and IV mRNAs in human optic nerve head by in situ hybridization. Investig. Ophthalmol. Vis. Sci..

[24] Lukas T.J., Miao H., Chen L., Riordan S.M., Li W., Crabb A.M., Wise A., Du P., Lin S.M., Hernandez M.R. (2008). Susceptibility to glaucoma: Differential comparison of the astrocyte transcriptome from glaucomatous African American and Caucasian American donors. Genome Biol..

[25] Howell G.R., Macalinao D., Sousa G., Walden M., Soto I., Kneeland S.C., Barbay J.M., King B., Marchant J.K., Hibbs M. (2011). Molecular clustering identifies complement and endothelin induction as early events in a mouse model of glaucoma. J. Clin. Investig..

[26] Johnson E.C., Jia L., Cepurna W.O., Doser T.A., Morrison J.C. (2007). Global Changes in Optic Nerve Head Gene Expression after Exposure to Elevated Intraocular Pressure in a Rat Glaucoma Model. Investig. Ophthalmol. Vis. Sci..

[27] Nikolskaya T., Nikolsky Y., Serebryiskaya T., Zvereva S., Sviridov E., Dezso Z., Rahkmatulin E., Brennan R.J., Yankovsky N., Bhattacharya S.K. (2009). Network analysis of human glaucomatous optic nerve head astrocytes. BMC Med. Genom..

[28] Pena J.D., Taylor A.W., Ricard C.S., Vidal I., Hernandez M.R. (1999). Transforming growth factor beta isoforms in human optic nerve heads. Br. J. Ophthalmol..

[29] Zode G.S., Sethi A., Brun-Zinkernagel A.M., Chang I.F., Clark A.F., Wordinger R.J. (2011). Transforming growth factor-beta2 increases extracellular matrix proteins in optic nerve head cells via activation of the Smad signaling pathway. Mol. Vis..

[30] Fuchshofer R., Birke M., Welge-Lussen U., Kook D., Lutjen-Drecoll E. (2005). Transforming growth factor-beta 2 modulated extracellular matrix component expression in cultured human optic nerve head astrocytes. Invest. Ophthalmol. Vis. Sci..

[31] Neumann C., Yu A., Welge-Lüssen U., Lutjen-Drecoll E., Birke M. (2008). The Effect of TGF- 2 on Elastin, Type VI Collagen, and Components of the Proteolytic Degradation System in Human Optic Nerve Astrocytes. Investig. Opthalmol. Vis. Sci..

[32] Schneider M., Pawlak R., Weber G.R., Dillinger A.E., Kuespert S., Iozzo R.V., Quigley A.A., Ohlmann A., Tamm E.R., Fuchshofer R. (2021). A novel ocular function for decorin in the aqueous humor outflow. Matrix Biol..

[33] Reed C.C., Iozzo R.V. (2002). The role of decorin in collagen fibrillogenesis and skin homeostasis. Glycoconj. J..

[34] Robinson K.A., Sun M., Barnum C.E., Weiss S.N., Huegel J., Shetye S.S., Lin L., Saez D., Adams S.M., Iozzo R.V. (2017). Decorin and biglycan are necessary for maintaining collagen fibril structure, fiber realignment, and mechanical properties of mature tendons. Matrix Biol..

[35] Iozzo R.V., Buraschi S., Genua M., Xu S.-Q., Solomides C.C., Peiper S.C., Gomella L.G., Owens R.C., Morrione A. (2011). Decorin Antagonizes IGF Receptor I (IGF-IR) Function by Interfering with IGF-IR Activity and Attenuating Downstream Signaling*. J. Biol. Chem..

[36] Iozzo R.V., Chakrani F., Perrotti D., McQuillan D.J., Skorski T., Calabretta B., Eichstetter I. (1999). Cooperative action of germ-line mutations in decorin and p53 accelerates lymphoma tumorigenesis. Proc. Natl. Acad. Sci. USA.

[37] Iozzo R.V., Moscatello D.K., McQuillan D.J., Eichstetter I. (1999). Decorin Is a Biological Ligand for the Epidermal Growth Factor Receptor. J. Biol. Chem..

[38] Vial C., Gutiérrez J., Santander C., Cabrera D., Brandan E. (2011). Decorin Interacts with Connective Tissue Growth Factor (CTGF)/CCN2 by LRR12 Inhibiting Its Biological Activity. J. Biol. Chem..

[39] Bredrup C., Knappskog P.M., Majewski J., Rødahl E., Boman H. (2005). Congenital Stromal Dystrophy of the Cornea Caused by a Mutation in the Decorin Gene. Investig. Opthalmol. Vis. Sci..

[40] Rødahl E., Van Ginderdeuren R., Knappskog P.M., Bredrup C., Boman H. (2006). A Second Decorin Frame Shift Mutation in a Family With Congenital Stromal Corneal Dystrophy. Am. J. Ophthalmol..

[41] Nikhalashree S., Karthikkeyan G., George R., Shantha B., Vijaya L., Ratra V., Sulochana K.N., Coral K. (2019). Lowered Decorin With Aberrant Extracellular Matrix Remodeling in Aqueous Humor and Tenon’s Tissue From Primary Glaucoma Patients. Investig. Opthalmol. Vis. Sci..

[42] Overby D., Zhou E.H., Vargas-Pinto R., Pedrigi R.M., Fuchshofer R., Braakman S., Gupta R., Perkumas K.M., Sherwood J.M., Vahabikashi A. (2014). Altered mechanobiology of Schlemm’s canal endothelial cells in glaucoma. Proc. Natl. Acad. Sci. USA.

[43] Sun D., Lye-Barthel M., Masland R.H., Jakobs T.C. (2009). The morphology and spatial arrangement of astrocytes in the optic nerve head of the mouse. J. Comp. Neurol..

[44] Simpson J.E., Ince P.G., Shaw P.J., Heath P.R., Raman R., Garwood C.J., Gelsthorpe C., Baxter L., Forster G., Ageing Neuropathology Study Group (2011). Microarray analysis of the astrocyte transcriptome in the aging brain: Relationship to Alzheimer’s pathology and APOE genotype. Neurobiol. Aging.

[45] Schneider M. (2017). The Role of Decorin in the Pathogenesis of Primary Open Angle Glaucoma.

[46] Schönherr E., Sunderkötter C., Iozzo R., Schaefer L. (2005). Decorin, a Novel Player in the Insulin-like Growth Factor System. J. Biol. Chem..

[47] Fukuchi T., Ueda J., Hanyu T., Abe H., Sawaguchi S. (2001). Distribution and expression of transforming growth factor-beta and platelet-derived growth factor in the normal and glaucomatous monkey optic nerve heads. Jpn. J. Ophthalmol..

[48] Kompass K.S., A Agapova O., Li W., Kaufman P.L., A Rasmussen C., Hernandez M.R. (2008). Bioinformatic and statistical analysis of the optic nerve head in a primate model of ocular hypertension. BMC Neurosci..

[49] Quigley H.A., Pitha I.F., Welsbie D.S., Nguyen C., Steinhart M., Nguyen T.D., Pease M., Oglesby E.N., Berlinicke C.A., Mitchell K.L. (2015). Losartan Treatment Protects Retinal Ganglion Cells and Alters Scleral Remodeling in Experimental Glaucoma. PLoS ONE.

[50] Rogers R.S., Dharsee M., Ackloo S., Sivak J., Flanagan J.G. (2012). Proteomics Analyses of Human Optic Nerve Head Astrocytes Following Biomechanical Strain. Mol. Cell. Proteom..

[51] Kirwan R.P., Wordinger R., Clark A.F., O’Brien C.J. (2009). Differential global and extra-cellular matrix focused gene expression patterns between normal and glaucomatous human lamina cribrosa cells. Mol. Vis..

[52] Kahari V.M., Larjava H., Uitto J. (1991). Differential regulation of extracellular matrix proteoglycan (PG) gene expression. Transforming growth factor-beta 1 up-regulates biglycan (PGI), and versican (large fibroblast PG) but down-regulates decorin (PGII) mRNA levels in human fibroblasts in culture. J. Biol. Chem..

[53] Roughley P.J., Melching L.I., Recklies A.D. (1994). Changes in the expression of decorin and biglycan in human articular cartilage with age and regulation by TGF-beta. Matrix Biol..

[54] Takeuchi Y., Matsumoto T., Ogata E., Shishiba Y. (1993). Effects of transforming growth factor beta 1 and L-ascorbate on synthesis and distribution of proteoglycans in murine osteoblast-like cells. J. Bone Miner. Res..

[55] Quillen S., Schaub J., Quigley H., Pease M., Korneva A., Kimball E. (2020). Astrocyte responses to experimental glaucoma in mouse optic nerve head. PLoS ONE.

[56] Johnson E.C., Morrisonab J.C., Farrell S., Deppmeier L., Moore C., McGinty M. (1996). The Effect of Chronically Elevated Intraocular Pressure on the Rat Optic Nerve Head Extracellular Matrix. Exp. Eye Res..

[57] Sawaguchi S., Yue B.Y., Fukuchi T., Abe H., Suda K., Kaiya T., Iwata K. (1999). Collagen fibrillar network in the optic nerve head of normal monkey eyes and monkey eyes with laser-induced glaucoma–A scanning electron microscopic study. Curr. Eye Res..

[58] Pijanka J.K., Markov P.P., Midgett D., Paterson N., White N., Blain E.J., Nguyen T.D., Quigley H.A., Boote C. (2019). Quantification of collagen fiber structure using second harmonic generation imaging and two-dimensional discrete Fourier transform analysis: Application to the human optic nerve head. J. Biophotonics.

[59] Zhu Y., Pappas A.C., Wang R., Seifert P., Sun D., Jakobs T.C. (2018). Ultrastructural Morphology of the Optic Nerve Head in Aged and Glaucomatous Mice. Investig. Opthalmol. Vis. Sci..

[60] Scott J.E. (1996). Proteodermatan and Proteokeratan Sulfate (Decorin, Lumican/Fibromodulin) Proteins Are Horseshoe Shaped. Implications for Their Interactions with Collagen†. Biochemistry.

[61] Weber I.T., Harrison R., Iozzo R.V. (1996). Model Structure of Decorin and Implications for Collagen Fibrillogenesis. J. Biol. Chem..

[62] Ling Y.T.T., Pease M.E., Jefferys J.L., Kimball E.C., Quigley H.A., Nguyen T.D. (2020). Pressure-Induced Changes in Astrocyte GFAP, Actin, and Nuclear Morphology in Mouse Optic Nerve. Investig. Opthalmol. Vis. Sci..

[63] Lu Y., Iandiev I., Hollborn M., Körber N., Ulbricht E., Hirrlinger P.G., Pannicke T., Wei E., Bringmann A., Wolburg H. (2011). Reactive glial cells: Increased stiffness correlates with increased intermediate filament expression. FASEB J..

[64] Yamaguchi Y., Mann D.M., Ruoslahti E. (1990). Negative regulation of transforming growth factor-beta by the proteoglycan decorin. Nature.

[65] Garwood C., Ratcliffe L.E., Morgan S.V., Simpson J.E., Owens H., Vazquez-Villaseñor I., Heath P.R., Romero I., Ince P.G., Wharton S.B. (2015). Insulin and IGF1 signalling pathways in human astrocytes in vitro and in vivo; characterisation, subcellular localisation and modulation of the receptors. Mol. Brain.

[66] Schaefer L., Tsalastra W., Babelova A., Baliova M., Minnerup J., Sorokin L., Gröne H.-J., Reinhardt D., Pfeilschifter J., Iozzo R. (2007). Decorin-Mediated Regulation of Fibrillin-1 in the Kidney Involves the Insulin-Like Growth Factor-I Receptor and Mammalian Target of Rapamycin. Am. J. Pathol..

[67] Annes J.P., Munger J.S., Rifkin D.B. (2003). Making sense of latent TGFbeta activation. J. Cell Sci..

[68] Flügel-Koch C., Ohlmann A., Fuchshofer R., Welge-Lüssen U., Tamm E.R. (2004). Thrombospondin-1 in the trabecular meshwork: Localization in normal and glaucomatous eyes, and induction by TGF-β1 and dexamethasone in vitro. Exp. Eye Res..

[69] Dietz H.C., Cutting C.R., Pyeritz R.E., Maslen C.L., Sakai L.Y., Corson G.M., Puffenberger E., Hamosh A., Nanthakumar E.J., Curristin S.M. (1991). Marfan syndrome caused by a recurrent de novo missense mutation in the fibrillin gene. Nat. Cell Biol..

[70] Dietz H.C., Pyeritz R.E., Hall B.D., Cadle R.G., Hamosh A., Schwartz J., Meyers D.A., Francomano C.A. (1991). The Marfan syndrome locus: Confirmation of assignment to chromosome 15 and identification of tightly linked markers at 15q15-q21.3. Genomics.

[71] Faivre L., Collod-Beroud G., Loeys B., Child A., Binquet C., Gautier E., Callewaert B., Arbustini E., Mayer K., Arslan-Kirchner M. (2007). Effect of Mutation Type and Location on Clinical Outcome in 1,013 Probands with Marfan Syndrome or Related Phenotypes and FBN1 Mutations: An International Study. Am. J. Hum. Genet..

[72] Conery A.R., Cao Y., Thompson E.A., Townsend C.M., Ko T.C., Luo K. (2004). Akt interacts directly with Smad3 to regulate the sensitivity to TGF-beta induced apoptosis. Nat. Cell. Biol..

[73] Danielson K.G., Baribault H., Holmes D.F., Graham H., Kadler K., Iozzo R.V. (1997). Targeted Disruption of Decorin Leads to Abnormal Collagen Fibril Morphology and Skin Fragility. J. Cell Biol..

[74] Vandesompele J., De Preter K., Pattyn F., Poppe B., Van Roy N., De Paepe A., Speleman F. (2002). Accurate normalization of real-time quantitative RT-PCR data by geometric averaging of multiple internal control genes. Genome Biol..

[75] Fuchshofer R., Yu A.H.L., Welge-Lu¨ssen U., Tamm E.R. (2007). Bone Morphogenetic Protein-7 Is an Antagonist of Transforming Growth Factor-β2 in Human Trabecular Meshwork Cells. Investig. Opthalmol. Vis. Sci..

